# Up in the air: Untethered Factors of Auxin Response

**DOI:** 10.12688/f1000research.7492.1

**Published:** 2016-02-03

**Authors:** Samantha K. Powers, Lucia C. Strader

**Affiliations:** 1 Department of Biology, Washington University in St. Louis, St. Louis, MO, 63130-4899, USA

**Keywords:** auxin, cell division, gene

## Abstract

As a prominent regulator of plant growth and development, the hormone auxin plays an essential role in controlling cell division and expansion. Auxin-responsive gene transcription is mediated through the TRANSPORT INHIBITOR RESPONSE1/AUXIN SIGNALING F-BOX (TIR1/AFB) pathway. Roles for TIR1/AFB pathway components in auxin response are understood best, but additional factors implicated in auxin responses require more study. The function of these factors, including S-Phase Kinase-Associated Protein 2A (SKP2A), SMALL AUXIN UP RNAs (SAURs), INDOLE 3-BUTYRIC ACID RESPONSE5 (IBR5), and AUXIN BINDING PROTEIN1 (ABP1), has remained largely obscure. Recent advances have begun to clarify roles for these factors in auxin response while also raising additional questions to be answered.

## Introduction

The plant hormone auxin plays a vital role in nearly every aspect of plant growth and development ^1^. Auxin-responsive gene expression relies on the TRANSPORT INHIBITOR RESPONSE1/AUXIN SIGNALING F-BOX (TIR1/AFB) pathway to trigger the expression of genes controlling auxin-regulated cell division, expansion, and differentiation ^2, 3^. Whereas the role of the TIR1/AFB pathway in the auxin signal transduction pathway has been well established, the existence of additional components raises the possibility that we have yet to uncover the entire story of auxin signaling.

**Figure 1.**
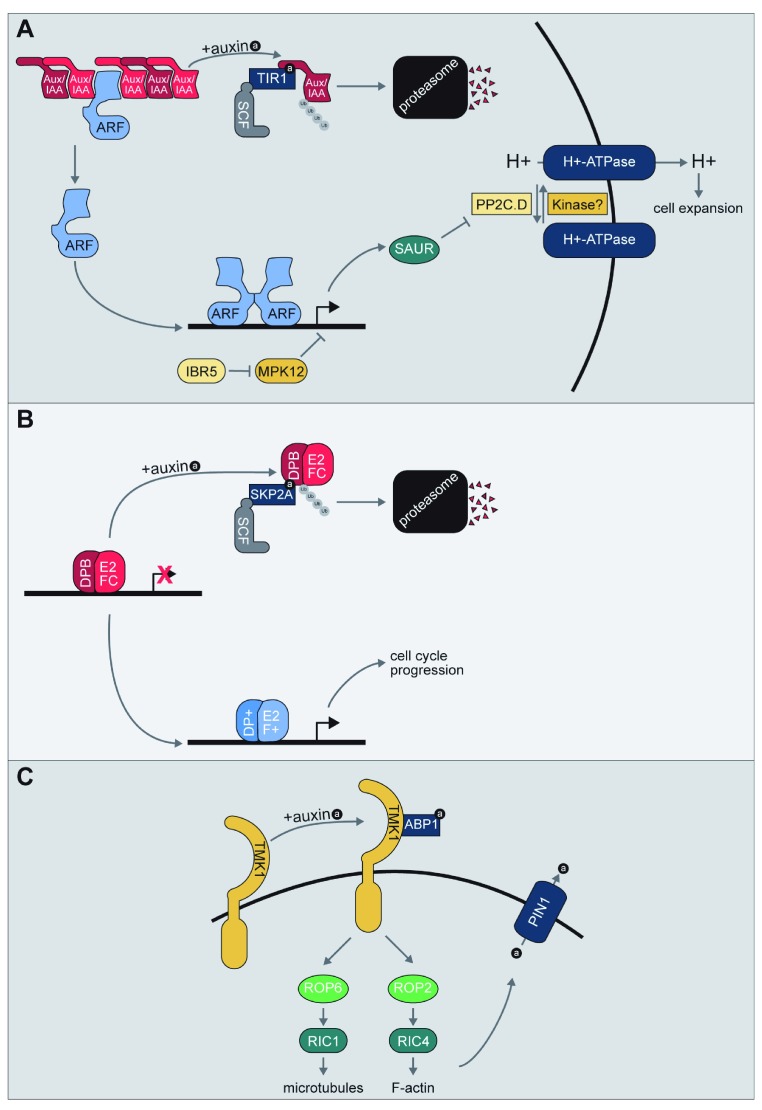
Auxin signal transduction pathways. ( **A**) Model of the TRANSPORT INHIBITOR RESPONSE1/AUXIN SIGNALING F-BOX (TIR1/AFB) signaling pathway. Auxin promotes the formation of the TIR1/AFB Auxin/INDOLE-3-ACETIC ACID INDUCIBLE (Aux/IAA) co-receptor to promote the ubiquitylation and subsequent degradation of the Aux/IAA repressor. Aux/IAA degradation relieves repression of AUXIN RESPONSE FACTOR (ARF) transcription factors, allowing for auxin-responsive gene expression. One of the transcript families upregulated by auxin is the *SAUR* family. The small SMALL AUXIN UP RNA (SAUR) proteins encoded by these transcripts have been suggested to play roles in multiple processes, one of which is interaction with and inhibition of members of the PP2C.D family of phosphatases, which act to regulate H ^+^-ATPase activity. Further, INDOLE-3-BUTYRIC ACID RESPONSE5 (IBR5) and MITOGEN-ACTIVATED PROTEIN KINASE12 (MPK12) have been implicated in regulating auxin-responsive gene transcription; this regulation is not through destabilization of the Aux/IAA repressors, suggesting a yet-to-be discovered mechanism of regulating auxin-responsive gene expression. For in-depth reviews of the TIR/AFB signaling pathway, please refer to 1, 3. For an in-depth review of SAUR proteins, please refer to 15. ( **B**) Model of the S-PHASE KINASE ASSOCIATED PROTEIN 2A (SKP2A) signaling pathway. E2FC/DPB repress expression of cell cycle genes. The F-box protein SKP2A binds auxin and promotes degradation of E2FC/DPB in an auxin-dependent manner. Degradation of E2FC/DPB relieves repression of cell cycle genes and allows for binding by activating E2F+/DP+ complexes. For an in-depth review of the SKP2A signaling pathway, please refer to 8. ( **C**) Model of the putative AUXIN BINDING PROTEIN1 (ABP1) signaling pathway. Apoplastic auxin is bound by ABP1, which allows for interaction with the TRANSMEMBRANE KINASE RECEPTOR (TMK) family of leucine-rich repeat receptor-like kinases. ABP1 binding of auxin regulates RHO-LIKE GTPASE 2 (ROP2) and ROP6 activation and binding to ROP interactive CRIB motif-containing proteins (RICs) proteins to positively regulate microtubule polymerization and F-actin polymerization and also alter PINFORMED1 protein localization to alter auxin efflux. For an in-depth review of the ROP/RIC system, please refer to 54.

Auxin signaling through the TIR1/AFB pathway involves three major protein families ( Figure 1A) – the auxin-binding TIR1/AFB F-box proteins, the AUXIN RESPONSE FACTOR (ARF) transcription factors, and the AUXIN/INDOLE-3-ACETIC ACID INDUCIBLE (Aux/IAA) repressor proteins ^2, 3^. In the absence of auxin, the Aux/IAA proteins repress activity of the ARF transcription factors ^4^. In the presence of auxin, the TIR1/AFB F-box proteins, which participate in a SCF (Skp1-Cullin-F-box) E3 ubiquitin ligase, interact with Aux/IAA repressor proteins to form a co-receptor, with auxin acting as the “molecular glue” ^4– 6^. This interaction results in ubiquitylation and consequent degradation of the Aux/IAA repressor proteins through the 26S proteasome, relieving repression of the ARF transcription factors and allowing for auxin-regulated gene transcription ^7^. Interactions among these three protein families is now understood at a molecular level ^2^ and provides a signal transduction pathway that controls auxin-responsive gene transcription in plants. For recent reviews of the TIR1/AFB pathway, please see 1, 3.

In addition to components of the TIR1/AFB pathway, other factors implicated in auxin response have been identified – however, roles for these components in regulating auxin signaling are much less understood. Although insight into the function of some of these factors, such as S-PHASE KINASE-ASSOCIATED PROTEIN 2A (SKP2A), SMALL AUXIN UP RNAs (SAURs), INDOLE 3-BUTYRIC ACID RESPONSE5 (IBR5), and AUXIN BINDING PROTEIN1 (ABP1), has been elusive, roles for these factors in regulating auxin outputs are slowly starting to be determined. In this commentary, we explore recent advances in our understanding of these factors and their roles in auxin response.

## SKP2A

Whereas extensive work has gone into understanding the auxin-binding capabilities of TIR1 and its downstream effects on auxin-regulated developmental responses ( Figure 1A), less is known about the molecular mechanisms that directly connect auxin to its role in cell division. Several cell cycle genes are upregulated by auxin ^8^ and other cell cycle genes contain auxin response elements in their promoter regions; however, these do not appear to be upregulated by auxin ^8^, suggesting that additional mechanisms linking auxin to cell division control may exist. Heterologously expressed SKP2A, an F-box protein involved in regulating the proteolysis of cell-cycle-related transcription factors ^9, 10^, directly binds auxin ^11^ and may provide a mechanism for auxin-mediated regulation of cell division ( Figure 1B).

The retinoblastoma-E2F pathway regulates the cell cycle through the interaction of E2F proteins with dimerization proteins (DPs) to form transcription factors that either activate or repress the expression of genes involved in cell cycle progression ^12^. SKP2A incorporates into an SCF complex ^9, 13^ with E3 ubiquitin ligase activity ^13^. Intriguingly, binding of auxin by SKP2A is necessary for degradation of E2FC and its dimerization partner DPB through the 26S proteasome ^11^. Degradation of E2FC and DPB relieves repression of cell cycle control genes to allow cell cycle progression ^9, 10^. In addition to regulating the degradation of E2FC/DPB, auxin binding promotes proteolysis of SKP2A itself ^13^, perhaps setting up a system in which auxin prevents SKP2A overfunction ^11^. The *skp2a* mutant hyperaccumulates E2FC and DPB protein ^10, 13^. Further, expressing a SKP2A variant unable to bind auxin in the mutant background fails to rescue this phenotype, suggesting that auxin binding by SKP2A is required for E2FC/DPB degradation ^11^. Overexpression of *SKP2A* results in increased cell division and induces lateral root primordia (LRP) formation, a process known to be dependent on auxin signaling ^9^. The molecular phenotypes of the *skp2a* mutant combined with the phenotypes of the *SKP2A* overexpression lines suggest a role for SKP2A in promoting auxin-regulated cell division.

Involvement of SKP2A in auxin binding and consequent degradation of the cell-cycle regulators E2FC and DPB implicate this F-box as a missing link connecting auxin regulation to cell division; however, relatively little is known about this pathway. Many questions remain for SKP2A roles in auxin-regulated cell division control. For example, SKP2A directly binds auxin and functions as part of an SCF complex responsible for targeting downstream components for degradation – are additional factors, other than E2FC and DPB, targeted for degradation by SKP2A to contribute to auxin response? Further, are activating E2F+/EP+ complexes similarly regulated by the proteasome? What is the effect on plant growth and development if this auxin-induced degradation of these factors is disrupted? There appear to be no gross effects on plant morphology in the *skp2a* mutant; is this because of redundancy or is it indicative of a minor role for SKP2A in plant growth and development? Answering these questions will inform our understanding of SKP2A roles in mediating auxin effects on cell division.

## SAUR proteins

Auxin regulation downstream of the TIR1/AFB pathway involves the induction of the early auxin response gene family *SAURs*. Initially discovered as auxin-induced transcripts in elongating soybean hypocotyls using a hybridization screen ^14^, multiple lines of evidence have been used to assign SAUR functions in auxin-related aspects of plant growth and development, including cell expansion, tropic growth, and apical hook development ^15^. Although SAURs represent the largest family of early auxin response genes, SAUR function in mediating auxin effects has only recently begun to be elucidated.

Ca ^2+^ is a well-known secondary messenger regulating developmental and physiological aspects of plant growth ^16^. Auxin has been proposed as a Ca ^2+^ activating signal ^17^; however, mechanisms connecting Ca ^2+^ to auxin signaling have remained largely elusive. SAUR proteins from multiple species interact with calmodulin (CaM) in a calcium-dependent manner ^18– 20^ and additional SAUR proteins are predicted to contain a CaM-binding site ^15^. Binding of SAUR70 to CaM or CaM-like proteins has been confirmed *in planta* ^19^; however, further studies will be necessary to determine the extent and functional relevance of SAUR-CaM interactions in auxin-regulated calcium signaling.

Several SAUR subfamilies, including SAUR19-24, are likely involved in cell expansion ^21^; however, mechanistic insight into SAUR roles in this auxin-regulated response has been lacking. Auxin has long been proposed to induce cell elongation through an acid growth mechanism ^22^, in which auxin is responsible for activating H ^+^-ATPases to acidify the extracellular matrix, in turn activating expansins and promoting solute and water uptake to drive cell expansion. Recently, Spartz *et al.* ^23^ determined that SAUR proteins promote phosphorylation of the C-terminal autoinhibitory domain of PM H ^+^-ATPases, causing activation. In addition, several SAUR proteins interact with a group of the D-clade PP2C phosphatases to inhibit phosphatase activity ^23, 24^. These phosphatases likely modulate the phosphorylation status of H ^+^-ATPases ^23^. Thus, SAURs likely act to inhibit the PP2C.D inhibitors of H ^+^-ATPase activity, suggesting a role for the SAUR proteins as positive effectors in auxin-mediated cell expansion through regulation of the PM H ^+^-ATPase activity. If PP2C.D deactivates H ^+^-ATPase activity through de-phosphorylation, it then follows that a kinase is necessary to activate H ^+^-ATPases. Many protein kinases have been proposed to regulate H ^+^-ATPase activity ^25^ – perhaps one of these functions in an auxin-dependent manner to regulate H ^+^-ATPases. Further, the identified SAUR-CaM interactions combined with the recently identified roles for SAUR proteins in regulating phosphatases raise the possibility that SAUR proteins could act as a link between calcium signaling, auxin, and phosphatase activity.

Recent studies have provided increasing evidence of SAURs’ importance in auxin-regulated plant growth and development. The large families of these proteins identified in widespread plant species suggest multiple and diverse SAUR functions in auxin response. One possible mechanism for SAUR regulation of such varied auxin-related plant responses may be provided by combinatorial diversity in SAUR-PP2C.D interactions. In Arabidopsis, there are 81 SAURs ^26^ and nine PP2C.D family members ^24^, many of which have been proposed to have differential expression patterns throughout the plant ^27^. Varying SAUR-PP2C.D combinations may regulate the phosphorylation status of distinct downstream elements to regulate different aspects of auxin response ^15^. Interaction experiments using the different SAUR-PP2C.D combinations may uncover whether SAUR proteins from additional clades also interact with these phosphatases. Further, the distinct subcellular localization and various developmental processes associated with individual SAUR proteins ^15^ suggest that SAUR targets in addition to H ^+^-ATPases likely exist. Whereas recent studies have finally begun to illuminate SAUR molecular functions in auxin response, further research will surely uncover additional mechanisms connecting the SAUR proteins to auxin signaling.

## IBR5 and MPK12

Auxin pathway roles for IBR5 and its interacting MAP kinase MPK12 remain enigmatic. The *ibr5* mutant was initially isolated for its resistance to the auxin precursor indole-3-butyric acid (IBA) ^28^, but subsequent studies revealed that *ibr5* was resistant to all tested auxins ^29^ and auxin transport inhibitors ^30^. In addition to reduced physiological responses to exogenous auxin application, *ibr5* mutants display developmental phenotypes ^29, 31, 32^ and reduced auxin-responsive transcription ^29– 32^, suggesting roles for IBR5 in the auxin signaling pathway.

*IBR5* encodes a dual-specificity protein phosphatase ^29^; related phosphatases de-phosphorylate MAP kinases ^33, 34^. Dual-specificity protein phosphatases are distinct from the PP2C-type phosphatases associated with SAUR activity (see above). IBR5 splice variants appear to play distinct roles in regulating plant growth and auxin responses and at least some of these roles may be independent of its catalytic activity ^31^. However, the IBR5 catalytic cysteine is necessary for auxin responsive inhibition of root elongation ^30, 31^ and IBR5 can de-phosphorylate the Arabidopsis MPK12 *in vitro* ^35^. Further, RNAi lines of MPK12 display auxin resistance ^35^, suggesting that IBR5 and MPK12 play opposing roles in regulating auxin responsiveness.

*ibr5* double mutants with other mutants that dampen auxin responses, including *tir1*, *axr1*, and *aux1*, exhibit additive auxin resistance in one or more bioassays ^30^. Most notably, combining *ibr5* with an auxin receptor mutant, *tir1*, greatly enhances auxin resistance relative to either parent ^30^, consistent with the possibility that IBR5 effects on auxin response are TIR1 independent. Similar to other auxin-resistant mutants, *ibr5* exhibits decreased levels of auxin-responsive transcripts ^29– 32^. However, unlike other characterized auxin-response mutants, Aux/IAA proteins are not stabilized in *ibr5* and are actually destabilized ^30, 31^, again suggesting that IBR5 modulates auxin signaling in a manner unique from other known auxin response regulators. ARF proteins mediate auxin-responsive gene transcription and the primary known mechanism of ARF regulation is repression by Aux/IAA proteins ^3^. The decreased auxin-responsive gene transcription ^29– 32^ combined with destabilized Aux/IAA repressor proteins ^30, 31^ observed in *ibr5* mutants suggest that either Aux/IAA destabilization is not the sole mechanism of regulating ARF activity or that there exists an additional auxin signaling pathway that ends in regulating the same gene targets as the TIR1/AFB pathway. Elucidating IBR5 and MPK12 targets may help differentiate between these possibilities. Recently, IBR5 was found to interact with SUPPRESSOR OF G2 ALLELE SKP1 (SGT1b), HEAT SHOCK PROTEIN90 (HSP90), and the Toll/interleukin-1 receptor domains of CHILLING SENSITIVE 3 (CHS3), SUPPRESSOR OF NPR1-1 (SNC1), and RESISTANT TO P. SYRINGAE 4 (RPS4) ^36^. IBR5 interaction with SGT1b may provide a mechanism for IBR5 regulation of auxin responses; mutants defective in *SGT1b*/ *ETA3* have been isolated as enhancers of *tir1* auxin resistance, perhaps by modulating 26S proteasome activity ^37^. SGT1b is a co-chaperone with HSP90. Further, TIR1 has recently been identified as a HSP90 client and HSP90 plays roles in integrating temperature and auxin signaling ^38^; perhaps IBR5 is an additional HSP90 client to allow temperature and auxin response integration.

## ABP1 and TMK1

Opinions about ABP1, first discovered over 40 years ago, have varied over the years. ABP1 has been proposed to act as an apoplastic auxin receptor whose downstream signal transduction pathway regulates cytoskeletal rearrangement and internalization of auxin transporters in response to auxin ^39– 41^. Study of roles for ABP1 in plant auxin signaling was limited by the reported embryo lethality of null *abp1* alleles ^42– 45^. In the absence of a viable allele, the field made progress in understanding ABP1 function by use of ABP1 knockdown lines, provided by expression of an inducible ABP1 antisense transcript or of an inducible single-chain fragment variable from a monoclonal antibody raised to ABP1 ^46^. The identification of an EMS-generated TILLING line, *abp1-5*, carrying a point mutation in the auxin-binding pocket of ABP1 ^47, 48^ allowed for intense study of ABP1 functions in auxin signaling and spurred an explosion of ABP1-related discoveries, uncovering roles for this elusive auxin-binding protein in a wide variety of processes throughout plant development ^39^. These new findings allowed for widespread acceptance of ABP1 as a *bona fide* auxin receptor ^41^.

The recognition of ABP1 as an auxin receptor, however, has once again been called into question. A recent report showing that new *abp1* null alleles display no obvious auxin-related or developmental phenotypes ^49^ was contradictory to earlier reports of the embryo lethality of the *abp1-1* null allele ^42– 45^ and the physiological and molecular phenotypes displayed by conditional knockdown and *abp1-5* alleles ^39^. This conflict was recently partially reconciled by the discovery that the embryo lethal phenotypes of *abp1-1* and *abp1-1s* were caused by a loss of the neighboring *BELAYA SMERT* gene rather than from loss of *ABP1* ^50, 51^ and that the *abp1-1* allele, which is null for *ABP1*, displays no obvious morphological phenotypes when the *BSM* defect is rescued in the mutant ^50^. Further, at least some of the phenotypes observed in the *abp1-5* TILLING allele may be the result of a background mutation in *PHYTOCHROME B* ^52^. Future work with new *ABP1* genetic resources ^49, 51^ will be necessary to clarify the role of ABP1 in auxin signaling and plant development and will determine whether *abp1* null alleles display molecular phenotypes.

The auxin-binding affinity of ABP1 combined with its widespread conservation throughout plants would suggest an important role for ABP1; however, lack of physiological phenotypes in the null mutants ^49, 50^ would suggest that ABP1 does not play a prominent role in Arabidopsis development. At this time, auxin-related roles for downstream components in the ABP1 pathway ( Figure 1C) including the TRANSMEMBRANE KINASE (TMK) family of receptor-like kinases ^53^ and the ROP-RIC system ^54^ remain unchallenged. This is a turbulent time in the ABP1 field as new discoveries are being made and roles (or lack thereof) for this signaling pathway in plant growth and development are being clarified. Roles for ABP1 in auxin response and development have once again become controversial; clearly more work will be needed to reconcile conflicting reports in this area.

## Conclusions and future directions

The existence of factors in addition to the components of the well-established TIR1/AFB pathway suggests that we have yet to uncover the entire story of auxin response. Recent advances in the field are slowly bringing to light roles in auxin signaling for each of these factors − SKP2A, the SAUR proteins, IBR5, and ABP1 – however, many questions still remain. In addition, although the TIR/AFB pathway appears to be well characterized, new structural data suggest that there are additional regulatory aspects of this pathway, including ARF proteins acting as molecular DNA calipers and ARF and Aux/IAA protein multimerization ^2, 55^ that have yet to be fully explored. Further research will undoubtedly uncover new regulatory mechanisms for the TIR/AFB pathway and molecular roles for these untethered factors in auxin signaling − the sky is the limit!

## Abbreviations

ABP1, AUXIN BINDING PROTEIN1; AFB, AUXIN SIGNALING F-BOX; ARF, AUXIN RESPONSE FACTOR; Aux/IAA, Auxin/INDOLE-3-ACETIC ACID INDUCIBLE; bHLH, basic Helix-Loop-Helix; CaM, Calmodulin; DP, Dimerization Protein; IBR5, INDOLE-3-BUTYRIC ACID RESPONSE5; MPK12, MITOGEN-ACTIVATED PROTEIN KINASE12; ROP, RHO-LIKE GTPASE; SAUR, SMALL AUXIN UP RNA; SCF, Skp1-Cullin-F-box; SKP2A, S-PHASE KINASE ASSOCIATED PROTEIN 2A; TIR1, TRANSPORT INHIBITOR RESPONSE1; TMK, TRANSMEMBRANE KINASE RECEPTOR.
